# Supplementary material for: Formula Graph Self‐Attention Network for Representation‐Domain Independent Materials Discovery
Source: Adv Sci (Weinh). 2022 Apr 27;9(18):2200164. doi: 10.1002/advs.202200164 (PMC9218748; doi:10.1002/advs.202200164)
Supplement: Supplementary file 1 — Supporting Information [file ADVS-9-2200164-s001.pdf]

Formula graph self-attention network for representation-domain independent materials discovery

Achintha Ihalage[1] and Yang Hao[1*]

[1]School of Electronic Engineering and Computer Science, Queen Mary University of London

[*]To whom correspondence should be addressed; E-mail: y.hao@qmul.ac.uk

Supporting Information

Figure S1: Distribution of train, validation and test data of all benchmark datasets.

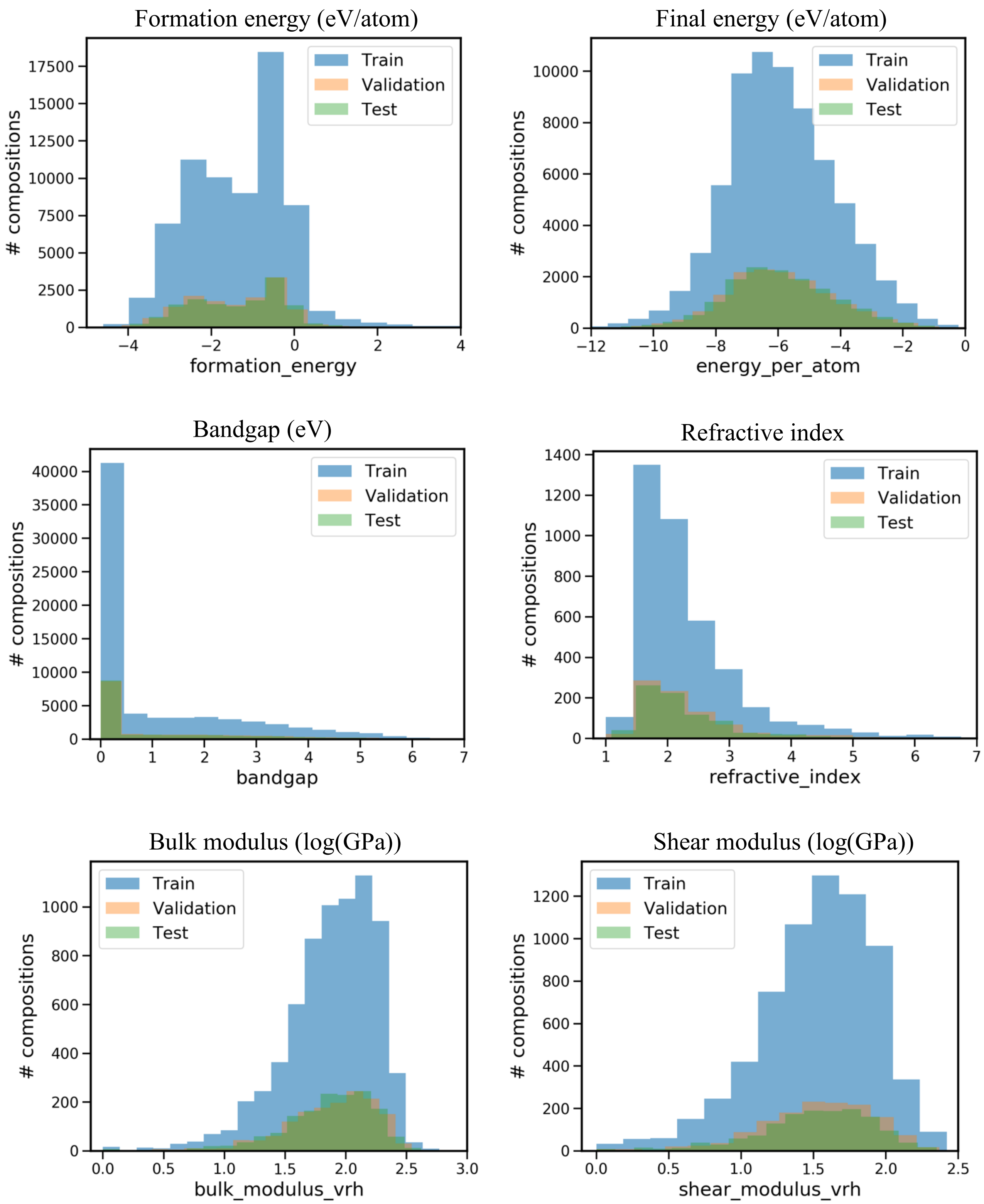

Figure S2: Training progress curves of Finder and Roost

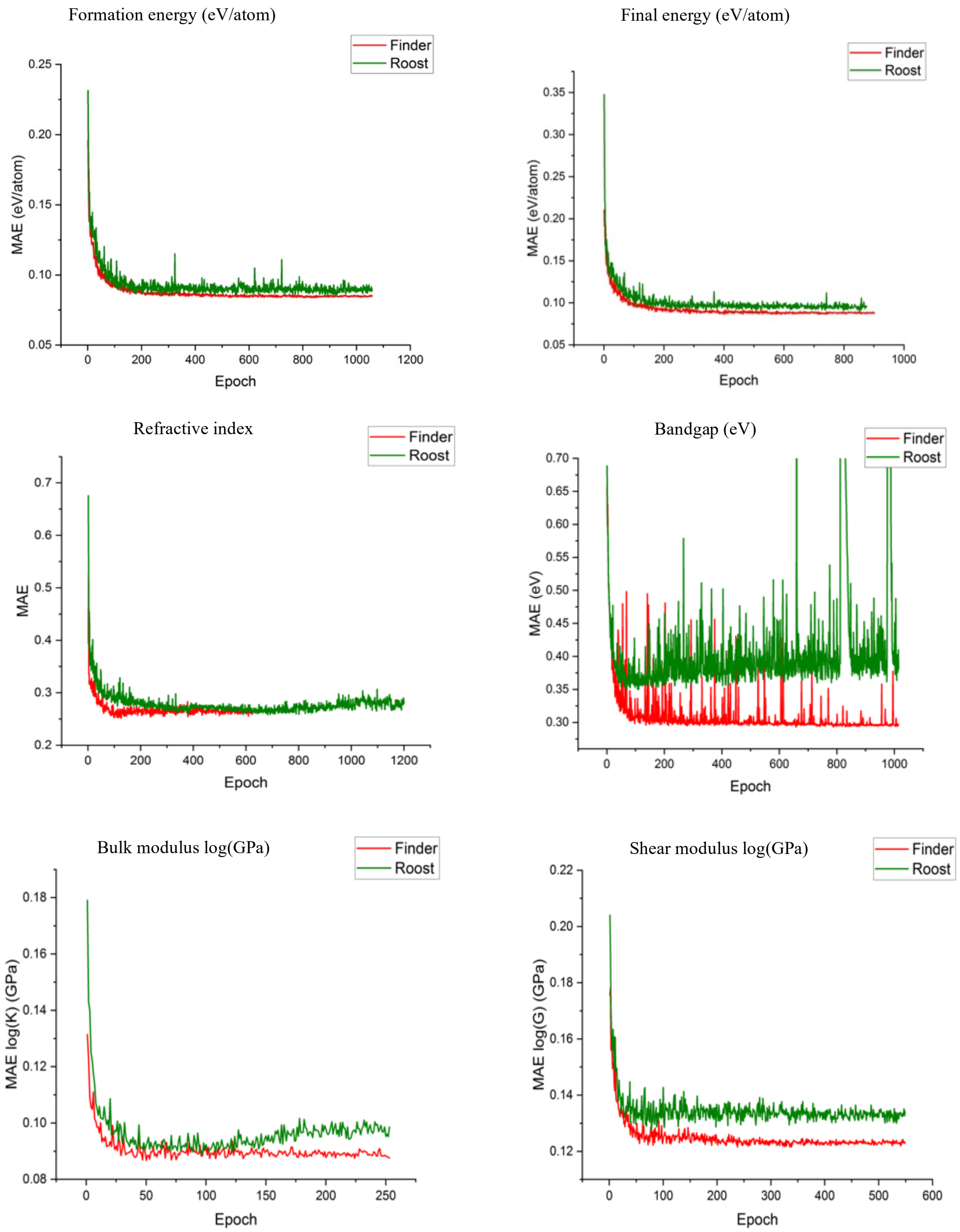

Figure S3: Parity plots of structure-agnostic Finder models

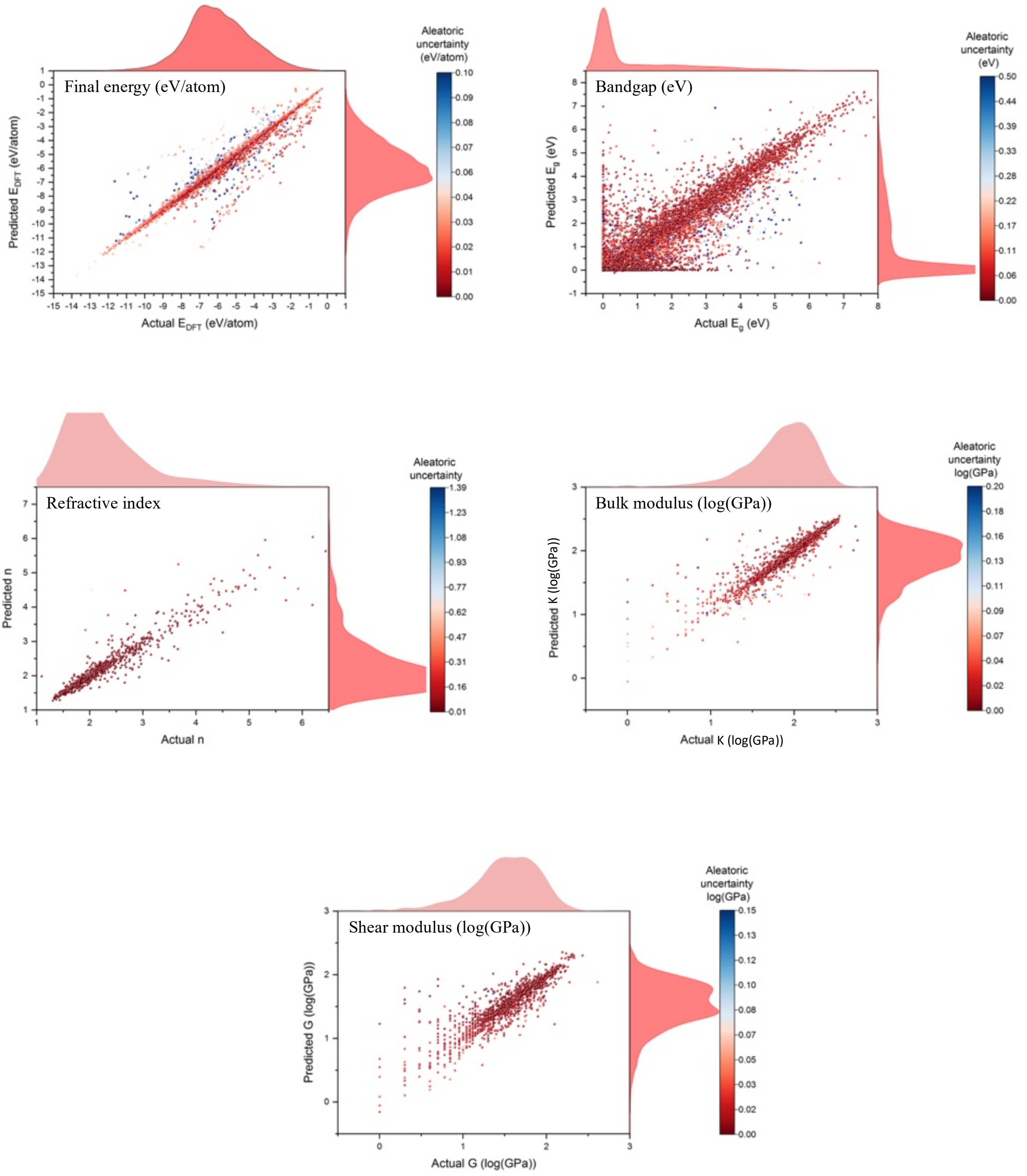

Figure S4: Parity plots of structure-based Finder models

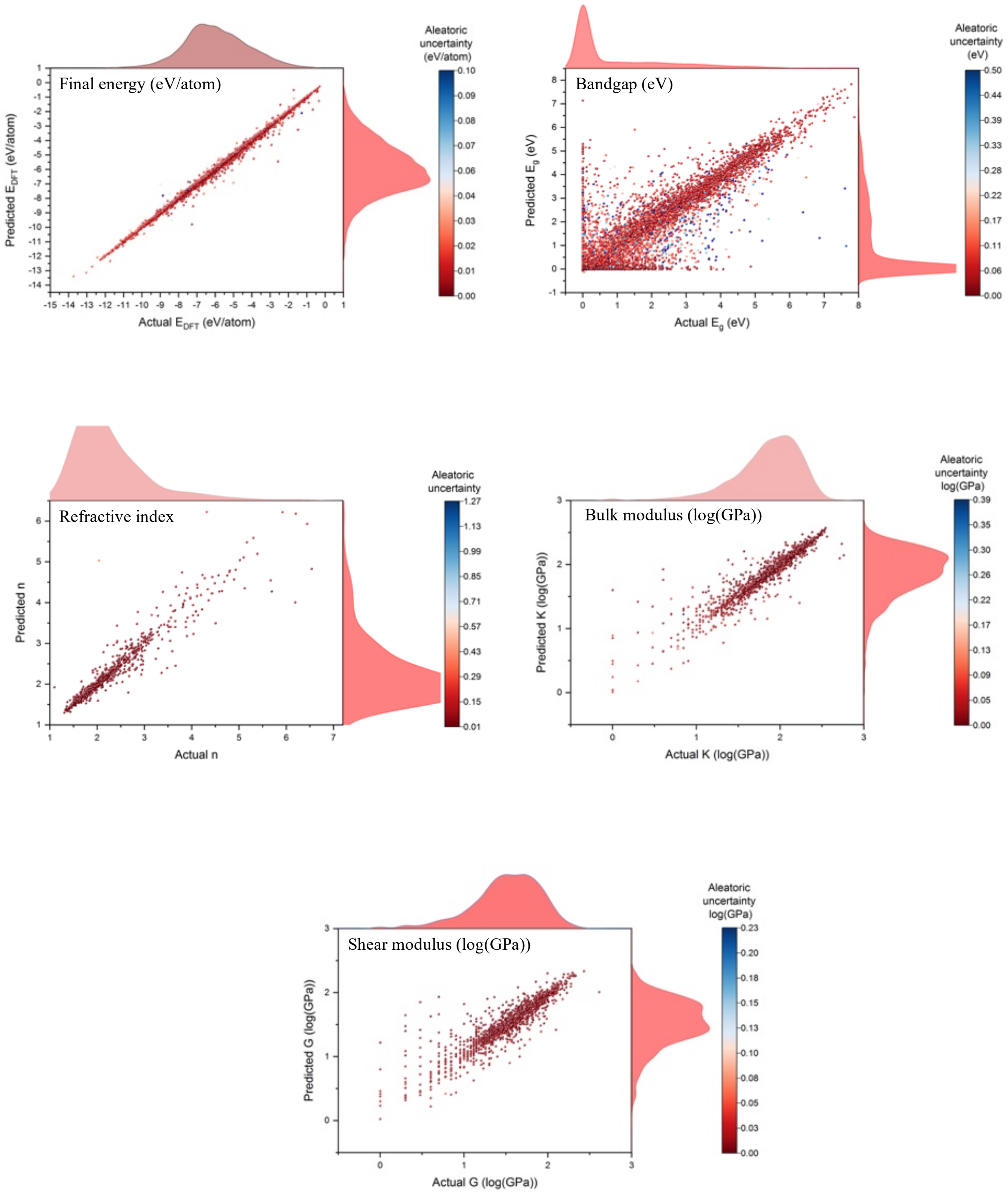

Figure S5: Principal component analysis (PCA) projections of the latent embeddings of crystals in the formation energy test database. The latent embeddings are taken from the final global attention pooling layer. Although structure-based model uses geometric information in the formula graph, both models produce similar material embeddings.

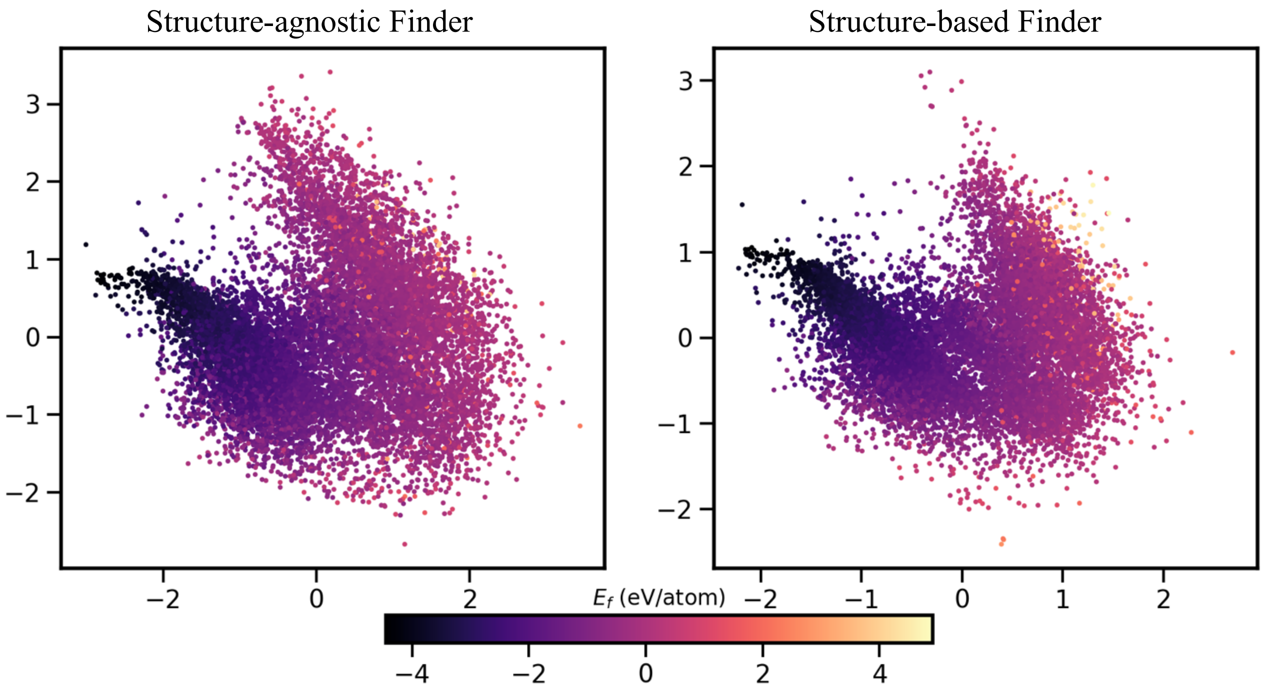

Table S1: Finder architecture details and parameters

Embedding type	Matscholar [1]
Embedding dimension	200
Internal dimension	200
Optimiser	Adam [2]
Learning rate	$3x10^{-4}$, reduced by a factor of 0.999 at every epoch
Batch size	128
Epochs	500 for structure-agnostic Finder and 1200 for structure-based Finder
Loss function	L_1 robust loss
# message passing layers	2
Output layer	Single unit or 3000 units for dielectric function prediction
Pooling operators	Δ_{agg} = 'element-wise mean' for local pooling within message passing layer. 'Soft-attention + element-wise sum' for global pooling
Distance expansion	Gaussian basis, 20 equidistant points between 0 and 5 with a std of 0.5 [3]
Batch normalisation	Only at message passing output layer.<br>mean=0, var=0.5, gamma=1, beta=0
Post processing neural network parameters	One convolutional-1D layer with 64 filters. 4 dense layers with 512, 1024, 1024 and 256 units. L_2 regularisation (10^{-6}) used to avoid overfitting.
Self-attention layer parameters (query, key and value networks and the internal dimensions)	d_K=d_Q=d_V=200, QNet, KNet and VNet each has a single layer with 200 units [4]

Table S2: ResCNN architecture details and parameters

Optimiser	Adam [2]
Learning rate	$3x10^{-4}$, fixed
Batch size	256
Epochs	500
Loss function	Mean absolute error (L_1)
Activation function	ReLU for hidden layers and linear activation at the output
Neural network architecture	Four convolutional 1D layers with filters 64, 128, 256 and 256, respectively → GlobalMaxpooling1D layer → Five dense layers with 256, 1024, 1024 and 2048, 512 units, respectively. Skip connections are added from leading convolutional layers to rear dense layers.<br>L_2 regularisation (10^{-4}) is used to avoid overfitting.
Composition features	Element fractions

References

[1] Vahe Tshitoyan, John Dagdelen, Leigh Weston, Alexander Dunn, Ziqin Rong, Olga Kononova, Kristin A. Persson, Gerbrand Ceder, and Anubhav Jain. Unsupervised word embeddings capture latent knowledge from materials science literature. *Nature*, 571(7763):95–98, Jul 2019.

[2] Diederik P. Kingma, Jimmy Ba Adam: A Method for Stochastic Optimization. https://arxiv.org/abs/1412.6980

[3] Chi Chen, Weike Ye, Yunxing Zuo, Chen Zheng, and Shyue Ping Ong. Graph networks as a universal machine learning framework for molecules and crystals. *Chemistry of Materials*, 31(9):3564–3572, May 2019.

[4] Ashish Vaswani, Noam Shazeer, Niki Parmar, Jakob Uszkoreit, Llion Jones, Aidan N. Gomez, Lukasz Kaiser, Illia Polosukhin Attention Is All You Need https://arxiv.org/abs/1706.03762
